# Understanding the sustainment of population health programmes from a whole-of-system approach

**DOI:** 10.1186/s12961-022-00843-0

**Published:** 2022-04-07

**Authors:** Melanie Crane, Nicole Nathan, Heather McKay, Karen Lee, John Wiggers, Adrian Bauman

**Affiliations:** 1 The Australian Prevention Partnership Centre, The Sax Institute, 235 Jones Street, Ultimo, NSW 2007 Australia; 2 Sydney School of Public Health, the University of Sydney, Sydney, NSW 2006 Australia; 3 Prevention Research Collaboration, Charles Perkins Centre, the University of Sydney, Sydney, NSW 2006 Australia; 4 Hunter New England Population Health, Hunter New England Local Health District, Wallsend, NSW Australia; 5 Hunter Medical Research Institute (HMRI), New Lambton, NSW Australia; 6 School of Medicine and Public Health, The University of Newcastle, Callaghan, NSW 2308 Australia; 7 National Centre of Implementation Science (NCOIS), The University of Newcastle, Callaghan, NSW Australia; 8 Department of Family Practice, University of British Columbia, 5950 University Boulevard, Vancouver, BC V6T 123 Canada; 9 Centre for Hip Health and Mobility, Vancouver Coastal Health Research Centre, 2635 Laurel St, Vancouver, BC V57 1M9 Canada

**Keywords:** Health promotion, Implementation, Sustainability, Population health, Systems thinking, Intervention, Noncommunicable diseases

## Abstract

**Background:**

Population health prevention programmes are needed to reduce the prevalence of chronic diseases. Nevertheless, sustaining programmes at a population level is challenging. Population health is highly influenced by social, economic and political environments and is vulnerable to these system-level changes. The aim of this research was to examine the factors and mechanisms contributing to the sustainment of population prevention programmes taking a systems thinking approach.

**Methods:**

We conducted a qualitative study through interviews with population health experts working within Australian government and non-government agencies experienced in sustaining public health programs at the local, state or national level (*n* = 13). We used a deductive thematic approach, grounded in systems thinking to analyse data.

**Results:**

We identified four key barriers affecting program sustainment: 1) short term political and funding cycles; 2) competing interests; 3) silo thinking within health service delivery; and 4) the fit of a program to population needs. To overcome these barriers various approaches have centred on the importance of long-range planning and resourcing, flexible program design and management, leadership and partnerships, evidence generation, and system support structures.

**Conclusion:**

This study provides key insights for overcoming challenges to the sustainment of population health programmes amidst complex system-wide changes.

**Supplementary Information:**

The online version contains supplementary material available at 10.1186/s12961-022-00843-0.

## Background

Reducing the population burden of chronic noncommunicable diseases (NCDs) requires prolonged investment in prevention [1], as changes in health behaviour or *outcomes* may only be evident after sustained delivery of population prevention programmes (PPPs). Sustainment in public health has been defined as “the sustained use or delivery of an intervention in practice following external implementation support” [2, 3]. This is understood to have occurred after full programme implementation is achieved and start-up funding is withdrawn [4]. Sustainment also largely depends on the nature of the intervention and outcomes [4, 5], as many population health changes may not be detectable until at least 3–10 years post-implementation [6].

Sustaining individual PPPs is a challenge. Effective programmes may evolve or be terminated, as funding, political or organizational priorities, or public support change over time [7, 8]. Termination may be an appropriate policy or resourcing decision, particularly if the programme is less effective delivered at scale, sometimes described as “voltage drop” [9, 10]. However, more often PPPs are terminated as a result of funding ending, or a lack of support from policy-makers, agency leaders or community [7]. If PPPs are terminated prematurely, they may fail to elicit individual health benefits [11] or positive return on investment [6].

Several frameworks and tools have been devised to identify or measure factors that facilitate or hinder sustainment of public health programmes [2, 9, 10, 12–14]. Others have been designed to assist decision-makers in assessing the sustainability of individual programmes [8, 15, 16]. These frameworks include intervention characteristics (e.g. components, adaptations, fit and cost), provider characteristics or inner context (e.g. funding, leadership support, champions and organizational capacity), processes (e.g. partnerships, communication, evaluation, planning) and the outer context (e.g. sociopolitical environments, polices and legislation) [3, 10, 12, 17]. The most prominent sustainability factors identified in the last 20 years of health promotion are organizational and delivery process factors [18]. However, these factors have been derived primarily from implementation studies of small-scale community-level settings [10, 19].

Less is known about how prevention programmes are sustained at the population level [4, 10, 20]. Population health focuses on population determinants and “system variables” that affect health [21, 22]. PPPs are programmes delivered at a city, state or national level, or other broad grouping, to affect health outcomes or health determinants distributed across the population [21]. These are relevant to policy-makers, and often require careful resource allocation. It is the complexity of public health problems, like obesity, which necessitate PPPs that use whole-of-system approaches to coordinate action across multiple levels, actors and agencies [23]. As “whole-of-system” programmes, PPPs are often facilitated by an overarching organization and a network of actors/agencies driving delivery (e.g. a statewide diabetes prevention programme delivered across the state population by local health service partners and community organizations) [24]. Thus, in PPPs, whole system-level factors come into play, introducing complex multiple intervening influences on delivery [12, 13, 25]. As such, what we know about linear influences of sustainment may no longer apply to PPPs [26]. In these circumstances, it is the context or *system dynamics* of what has been sustained and why, that is most important [27].

How interventions are sustained across the health system is a knowledge gap which we sought to address in this study. Our aim was to explore the factors and mechanisms contributing to sustainment of prevention programmes at this population level of intervention. Specifically, we explored the health system context for sustaining PPPs and the relationship between real-world experiences and current theoretical understanding. To guide the study, we applied a “systems thinking” lens. Systems thinking is an approach to understanding the behaviour of a complex system—“its elements, interconnections and purpose” [28]. Systems thinking has become an important framework for solving complex disease prevention issues [29–31]. Specifically, this involved looking at how the system for sustaining PPPs functions, the interconnected processes and the relationships that enable it to “run the way it does” [28, 32].

## Methods

### Context

Health promotion in Australia involves the health system, local municipalities, state and national government and nongovernmental organizations (NGOs), advocacy groups, professional bodies, universities, training and funding agencies. Each state system has different organizational structures to promote health. Some states, like Victoria and Western Australia, have established semi-independent state health promotion funding bodies (e.g. VicHealth, Healthways), while other states have embedded health promotion into the government service delivery structure, sometimes through partnerships with universities to create implementation laboratories [33] that implement and evaluate health-promoting activities.

### Study design, participants and recruitment

We undertook a qualitative study engaging practitioners and decision-makers in public health in Australia with experience in population health. We used purposive sampling techniques to recruit a range of prevention experts from diverse organizations and who worked at different levels within health promotion (i.e. local, state or national level). Using initial networks, we identified prominent prevention practitioners or decision-makers (stakeholders) within Australia, supplemented by other recommendations by initial participants. Eligible participants were selected based on having experience at the population level and/or working in a management position for more than one term of government (i.e. > 3 years), and whose role was to oversee the delivery of PPPs. Prospective participants were sent an information statement via email to consider participation and were called to confirm their interest and schedule the interview. Invitations to participate in the study continued until we reached a saturation of ideas.

### Data collection

One-on-one video interviews were conducted with consenting participants between June and August 2020 using Zoom software. All interviews were conducted by one experienced qualitative researcher (MC). Participants provided verbal or written consent before the interview was conducted. Interview were audio-recorded and took 52 minutes on average to complete.

We developed a semi-structured interview guide to examine the factors influencing PPP sustainment. We invited study participants to reflect on their experiences while managing, implementing or sustaining public health intervention(s) throughout their career. Participants were asked to consider PPPs that were/were not sustained as changes in government or government priorities or social, political or technological changes occurred. The interviewer took field notes during the interviews to inform the analysis and used follow-up questions to prompt the participant.

We developed our interview questions by using the Program Sustainability Assessment Tool (PSAT) as a guide. The PSAT is a quantitative instrument developed to assess the sustainability of public health programmes. The PSAT comprises eight domains/subscales of factors associated with the sustainability of public health programmes [15]. Interview questions were designed to explore the influence and importance of these domains with respect to PPPs the participant had managed, with a focus on how/whether disruption in the system implementation affected programme sustainment. We explored programme vulnerability to system changes, such as changes in government leadership or political priorities, or social changes, such as that recently created by the COVID-19 pandemic. The PSAT domains we assessed included the role of partnerships; social-political support from decision-makers and stakeholders; funding for ongoing programmes; strategic planning for sustaining implementation; organizational processes, learning and resilience; programme adaptations; the role of programme evaluation and research; and communication strategies; as well as soliciting general reflections on sustainment and key learnings (Additional file 1). Co-authors (MC, NN and KL) pilot-tested and refined all interview questions prior to the start of the study.

### Data analysis

We transcribed interview recordings verbatim and checked for accuracy. We applied thematic analysis methods using a deductive framework to explore the system dynamics of the data. Specifically, we considered the system context driving sustainment [34]. In applying a systems-thinking framework, we did not seek to explore the system “stock-and-flow” structures or rules [28], but the broader contextual functioning of the system contributing to sustainment. MC reviewed transcripts and recordings and made initial observations, then used line-by-line coding with descriptive words or phrases to describe data segments to develop an analytical framework of constructs from which to derive higher-level codes [35]. We compared differences between the various participant “actors”, their level in the system (local, state, national) and their role (decision-maker, government or nongovernmental health promotion practitioner, researcher) and their interactions. MC reviewed initial coding and emerging themes with co-authors (NN and KL). Key themes and interpretations were then discussed with all co-authors as a process to establish theoretical notes. We reflected on the themes that emerged from our experience managing PPPs or influencing health promotion practice at some level or capacity and considered the correlation of findings with the existing theoretical base through the PSAT. All participants responded to the invitation to check qualitative themes and provide feedback. Participants affirmed the findings, and some provided further examples or detail on their understanding of state and local differences. We used NVivo software (QSR International Pty Ltd. version 12, 2018) to code and explore analysis.

## Results

### Participant characteristics

We identified 14 eligible stakeholders. At the recruitment stage, one eligible stakeholder did not respond when contacted. All stakeholders who did respond agreed to participate in an interview. In total, we conducted 13 interviews with stakeholders who were governmental (*n* = 7) and nongovernmental (*n* = 6) health promotion experts. They held senior leadership (*n* = 6) or mid-management (*n* = 7) roles within their organization. Many participants had experience in both government and NGOs, and all had been in public health for at least 10 years. Four participants had a position with a research focus or adjunct academic position. Most participants were female (*n* = 10), and most were from the state of New South Wales (NSW) (*n* = 8).

### Key barriers to sustaining population prevention programmes

From the exploration of factors, we identified four key barriers to the sustainment of PPPs: (1) short-term political and funding cycles, (2) competing interests, (3) silo thinking within the health system delivery, and (4) population fit. We summarize these findings in Table 1.

**Table 1:** Summary of key barriers and corresponding enablers for sustaining population prevention programmes

*Barrier*	*Enabler*	*Summary description*
Short-term political and funding cycles	Long-range planning, evidence-building and partnering	Long-term planning in terms of strategic resourcing and internal funding reduces staff turnover and knowledge loss. Partnering with other organizations to help alleviate reduced resourcing burdens. Evidence of PPP impacts can help to support ongoing programme investment
Competing interests	Organizational/political leadership, public support, collaboration and strategic implementation	Communication channels with senior management and public to promote health promotion and PPP benefits. Working with other sectors at the policy level and partnering with end-user organizations to deliver programmes as routine
Silo thinking	System structure and supports	Using a systems approach to harness the support of the whole health system through support structures such as policies, strategy planning documents, service agreements, funding, infrastructure, communication channels and relationships
Programme fit in the population	Flexible programme delivery, broad focus, local adaptability and agility	Broad multicomponent PPPs at scale to withstand change and flexible to the changing contextual and local needs

*Note*: Table is presented as a summary but is not intended to show a linear relationship

#### Short-term political and funding cycles

Political and economic environments in which PPPs were implemented was highlighted by most as a fundamental population health challenge. A few of the participants explained that programmes designed to change health behaviours and reduce NCDs generally require a long-term programme effort. However, this was not achieved in practice. PPPs were most often planned for short-term implementation, supported by short-term funding (usually 3 years maximum) and measured against short-term outcomes. Thus, programmes had limited capacity to improve health in a cost-effective way. The implication of short-term programmes and funding cycles discussed by a few was that it generated staffing insecurity. In turn, a short-term workforce resulted in corporate memory loss. “Short-termism” was broadly discussed by participants working across the national and state level, in government and NGOs, where funding streams, grants and commitments were time-bound. One manager mentioned that this short-term approach was often used as a way to minimize potential risks to organization finances or reputation. However, they also stated that health promotion tended to be too risk-averse.

*Diet and obesity interventions are incredibly complex and they're multi-caused and there are different levels. So the interventions that are required are likely to be interventions that require many decades of intervention.* #11 State NGO manager

*The short-term funding cycles can actually breed, in some respects, bad practice. So, what you're actually developing and what you're looking to implement is influenced by the fact that you only have 3 years. You might go for low-hanging fruit, you might go for solutions that you might not have gone for if you knew you had a longer period of time. You don't learn from your mistakes. You don't get to evolve and massage and improve your initiative.* #13 National NGO senior manager

The political environment was a key contributor to programme sustainment. Participants spoke of the challenges in sustaining PPPs when government priorities changed. State government priorities dictate the direction of health services and public health. Publicly administered PPPs either adapt to fit with government priorities or risk being terminated. Termination was not necessarily viewed as a negative: half of the participants noted the need to end programmes that were unproductive, or “refresh them” to “sustain impact appropriately”.

NGOs could support programmes being continued despite new political priorities. However, priority changes often stretched public health funds and resources. Political interest in a health issue or programme was not always regarded favourably. Some participants were concerned that partisan programme popularity might reduce the likelihood of retaining support for a PPP should a change in government leadership occur.

A general response was to persevere through fluctuating political or social climate cycles and accept short incremental “wins”. Potentially longer-term funding, such as that provided by Australia’s National Partnership Agreement on Preventive Health (NPAPH), was mentioned by most participants as a major driver of PPP implementation—and may have been perceived as a stable environment for PPPs. (The NPAPH was established in 2008 with an investment of $873 million to prevent lifestyle-related risk factors for NCDs for state PPPs. It was abolished in 2014 with a change in government [36]. While there was some shock at sudden funding loss, those in senior leadership were not surprised by the transience of the NPAPH).

#### Competing interests

Given “scarce health resources”, competing for funding and resources with other priorities in health (or government ministries more broadly) was perceived as a constant challenge. Within health, PPPs competed with other public health and health service priorities with immediate or short-term visible impacts on health, having longer-term outcomes often beyond the tenure of senior health service executives or political leaders.

Participants recognized that senior leadership strongly influenced the extent to which health promotion was supported or sustained. Some participants spoke about the health promotion background or experience of current or former executives as influencing whether PPP received support.

*I think one of the things that can interfere is of course how much your executive are on board with health promotion, and that's certainly seen us with stronger or maybe sometimes the strategies are different, and I think for health promotion you really have to be constantly looking up ways to promote yourself.* #04 Local health manager

Competing priorities existed outside the health sector, as population health strategies to address health determinants often required actions outside the jurisdiction of health departments and health ministers. Influencing these meant engaging politicians with different priorities. One example was the need to work with a government office responsible for liquor licensing to reduce the health harm from alcohol use, whose responsibilities also intersect with other government priorities including the night-time economy. Participants in senior government positions noted a greater need to communicate with counterparts in other sectors to support public health programmes. Mature thinking about the role of other organizations in health promotion, and caution about the capacities and interests of other agencies, was also deemed important.

*We get caught up in our sense of what we're doing—health is the most important thing and that education should be doing this and transport should be doing that, etc. And I think it's that question about when you're working with other *people*, health's not their core business… But they've got their organizational priorities and they've got the things that they have to deliver.* #06 State government senior manager

#### Silo thinking within the health system

The sustainment of PPPs across the health system was described as “passing responsibility” to another organization or another part of an organization, as a way of programme institutionalization. This “downstream” approach was described by nearly half of participants as a function of ensuring sustainment, either from state to local government or from NGO to other community organizations. One state-level manager noted a common mindset in their organization that, as soon as fiscal responsibility for a PPP was transferred to the local health services department, state responsibility ended. Programmes were sustained by transferring programme costs and responsibility to other sectors such that the PPP was “embedded in a sector”. Such thinking came at the expense of value, in terms of the economies of scale and efficiency.

Implications of compartmentalized programme actions and activities was reflected in funding needs. Funding was discussed as either “critical” or “mildly necessary” to sustain PPPs. This view depended on the level that the person worked within the health system. Those in state oversight positions did not perceive funding as a barrier to sustaining PPPs. They looked to other mechanisms such as policies or partnerships to achieve programme sustainment, whereas those in local government health services or NGOs viewed funding as essential to sustaining PPPs. This strongly influenced the way these groups related to funders including state government. Four participants mentioned that innovations were risks that local-level government and NGOs were cautious about, as they could jeopardize potential funds.

*I think for NGOs it has to come down to funding. There has to be a commitment for funding if NGOs are to deliver something.* #10 State NGO manager

*Just don't do anything too flashy that can be aligned with one particular government or one particular minister, which is really hard because they want you to do that work because they want the profile. …big flashy things don't survive because when there’s a change—even with the new CEO [chief executive officer]—the new one wants to make their own mark. So that doesn't continue, the new stuff does.* #11 State NGO manager

Failure to recognize the role and value each part of the larger system required to implement and maintain large-scale programmes created tensions between levels of government and between government and NGOs. The local delivery managers felt that the state operating team generally did not understanding contextual issues that influenced the delivery of PPPs at the local level. This was said to result in unachievable key performance measures and programme outcomes in some areas.

*We started seeing, “this is the programme”, “these are the practices”. It became much more regimented around “this is what the programme looks like”, “these are the deliverables”, “this is the outcome we're looking for and you will be assessed … and whether they pass or fail are based on whether they meet the practices”… I was burning out staff trying to get this model to work.* #09 Local health senior manager

*If we're the local implementers, it should all sing together, we should be a band. We should be a very good band or orchestra, *whatever* you want to call us, different components… But down here we're probably all the ones making the—they might be the bigger sound, we're doing all the blending…* #04 Local health senior manager

#### Population fit

All the participants were managers responsible for a suite of PPPs, and it was widely accepted that no one PPP alone could effectively address population-level NCD prevention. Individual PPPs were viewed as a component of a wider population health commitment. Many participants spoke about the fact that sustaining health promotion was more critical, and of higher priority, than sustaining a specific PPP—which placed single programmes or programme elements at risk for defunding.

PPPs designed for a specific purpose were difficult to adapt to changing population needs or align with new priorities—this was mentioned by many of the participants in the context of the current COVID-19 crisis and lockdown measures, where many PPPs they managed were only designed to be delivered face-to-face. One participant explained that PPPs were generally developed with a specifically defined format and purpose. As such, some participants considered it necessary to retire those programmes. Other participants mentioned their caution about committing to long-term partnerships where they may be locked in to contracts and unable to adapt the programme. Regardless, some programmes [Cancer Council skin cancer prevention programme and the Heart Foundation’s Jump Rope for Heart] were successfully delivered over decades.

*We set them up initially to deliver against certain outcomes, and often there is radical changes, particularly in how people consume information, their lifestyle factors, even the people who come into the programme.* #05 State government manager

*Health promotion, it's never one single thing that it's going to make a difference. It’s all those things happening at once.* #10 State NGO manager

The delivery of PPPs at scale, directed by state- or national-level teams across local health services, offered advantages and disadvantages for sustainment. Many of the participants noted that localized small-scale PPPs were more easily terminated, whereas PPPs delivered at scale across local levels could better withstand political pressures, especially if they had a greater public profile and a central implementation structure. However, lack of local health service teams’ involvement in decisions on statewide PPPs was considered by local-level managers as a potential barrier to PPPs fitting local contexts. These participants asserted that their knowledge of local issues and capacity to adapt at-scale PPPs to their local population context would ensure not only implementation but also ongoing sustainment. From a resource management perspective, two senior managers noted that while resourcing of at-scale state-led programmes was more stable, it was critical for staff to spend time across more than one PPP to ensure job variety, and therefore staff retention.

*Because otherwise it's like that little thing of, “Well, that's just a little thing over here, and yeah, that'll be sad if that goes,"… But with some of these flagship programmes—I mean they can't all be big programmes, they all have to start somewhere as well—but these as flagship programmes … part of the reason why it was able to survive.* #06 State government senior manager

### Key enablers for sustaining population prevention programmes

Several approaches were used to overcome barriers to sustainment. We highlight these mechanisms as “enablers” and contextualize them in Table 1. However, most participants recognized that the ability to fully address barriers to sustainment required transformational changes in the system—including funding structures, performance measures, planning and design.

#### Long-range strategic planning and resourcing

Strategic planning was important particularly in the face of changeable political, economic and social climates. Most participants at the various levels spoke pragmatically about how they distributed and allocated health promotion staff and other resources to maximize the capacity to sustain multiple PPPs. Longer-range planning meant looking for partners to share responsibilities and invest in the PPP over time. Embedding PPPs into medium- and long-term strategic documents was also considered an important strategy to sustain PPPs within government organizations. Some participants mentioned using formalized partnerships with local agencies and community organizations to support PPPs. Service agreements were mentioned by some local and NGO managers as a mechanism to embed programme delivery into local health services. Key performance indicators for PPPs were mentioned by the state government managers to clearly identify programme deliverables. State and local government health managers viewed NGOs as important partners despite political changes and challenges. NGOs viewed themselves as more resilient to government disruption or change; they fostered bipartisan political relationships to remain flexible and sustain their programmes.

*In the NGO sector, where we are perhaps, we are subject to economic fluctuations and to some extent, changes of government because of government funding, but generally speaking we can make longer-term commitments because a change of government doesn't affect what we choose to prioritize.* #13 National NGO senior manager

*Having a commitment for whoever it is to deliver it—it doesn’t always have to be NGOs—but it needs to be a long-term commitment. And build in that evaluation so that at the end of the 4 years of funding …they [decision-makers] can see what realistic impacts it has had so that they can continue on. Because they might not see a decrease in prevalence within 4 years.* #10 State NGO manager

#### System support and structure

Most participants viewed population health from a “systems perspective”. That is, when they described their role or that of others, they spoke about it as part of a complex health system, with many levels and where different organizations contributed to PPP delivery and sustainment. This contrasted with “silo thinking”, as PPPs were discussed in terms of multilevel implementation by multilevel implementers.

This perspective was enabled by “system structures”, such as governance structures and processes, and local community setting services. System supports mentioned included policies, strategic planning documents, and service agreements and quality frameworks, sustainable funding, health promotion institutions and other mechanisms or “infrastructure scaffolding”. Intangible supports, such as relationships, were also mentioned by most as the key mechanism sustaining PPPs across levels. These supports seemed to be used to enable statewide PPPs to withstand the pressures of competing priorities, and improved the fit of PPPs to local contexts. State-level support to deliver a PPP across local municipal networks was mentioned by two local-level health service managers to make it easier for local-level health providers to allocate time and resources.

*What matters more than anything else is the systems that sit behind that intervention. So, to what extent are there robust medium- to long-term implementation plans, funded action plans, robust institutions …institutional and *governmental* systems—building blocks without which, or in the absence of which, sustainability is really difficult.* #13 National NGO senior manager

*So, for us, we implement state programmes, and with the funding that we get for those programmes, that is a sustainability strategy because it's funded…. The health promotion directors will also want it in writing … it's really important for us to use as evidence when we try to protect our budgets.* #04 Local health senior manager

#### Flexible design and management

While it was critiqued by some as not currently occurring or not occurring adequately, half of the participants suggested that evolving population health needs could be met through “inbuilt” capacity for programme adaptability. Flexibility was discussed by most as necessary in terms of how at-scale PPPs are designed and implemented to fit local contexts. This included how state-led programmes were delivered in local health services or how NGO or local health service programmes were delivered by smaller community organization partners. From a management perspective, one way of increasing flexibility while maintaining fidelity at scale within state-led programmes, as one state programme manager describes, was to use a “tight–loose–tight” approach (i.e. enabling “loose” adaptable programme activities within the confines of core “tight” activities). The local implementers also described that approach as essential because local populations and their needs differ. Flexibility provides the opportunity for testing different implementation approaches in specific contexts, without which programmes were likely to fail.

*There’s a point at which they need to be refreshed, or reinvigorated, or revised in some sort of constructive way.* #05 State government senior manager

*The funding goes to the health districts and they’re told what they need to deliver, that’s the “tight”. The “loose” is “do it as you see fit”, but obviously with a lot of support and involvement in the office. The “tight” is you now have to report on your performance indicators, and formally report on those.* #03 State government manager

#### Leadership

During a crisis (such as unexpected funding cuts), organizational and political leadership were highlighted by most as key factors that ensured a programme survived. Where political leadership support failed, public support for programmes also helped to influence decisions. Gaining a public profile and public support was important but viewed as a lower priority than building influential relationships and communicating programme benefits directly to senior decision-makers.

*The one at the top of my list would be high-level senior executive or political support. Without that, no matter how good an intervention is, it’s just not going to be sustained, particularly if we’re talking about government, but I think the same can apply working in not-for-profit.* #01 State NGO area manager

Relationships between programme managers and senior organizational leadership was regarded as critical to promoting specific PPP profiles and health prevention more broadly. Maintaining relationships with organizational leaders and defending programmes was considered both a necessary and resource-intensive communication activity.

#### Partnerships

Non-health partners were deemed necessary to sustain programmes when resources were limited. This was mentioned widely by the NGOs and government managers. It was noted that health may not be the “core business” of partner organizations, and they often had a limited understanding of health promotion. However, overcoming funding or resourcing challenges and reaching end users required the ability to readily partner with community organizations to deliver programmes over the longer term. Finding key people or champions in these organizations was thought to be important at state and local levels. However, relying on individual champions for programme sustainment was mentioned by a few as a risk for the programme’s effectiveness and longevity. Four participants stated that for health promotion to remain a priority, PPP actions or activities needed to be routinized, and to do this, organizations needed to clearly identify how PPPs aligned with end-user goals.

*There's no point us *inventing* something that no one can pick up and is not scalable and can't be funded. So I've got a target agency to make my intervention routine practice…the answer is you design it with your end user, the end-provider organization in mind.* #07 Local health senior manager

*I think the most crucial thing is whatever we are developing in terms of an intervention needs to be part of whatever organization is delivering it, part of their core business. It’s just an adaptation of what they do. If it's too different, then there’s no sustainability.* #10 State NGO manager

#### Research and evaluation evidence

Research evidence was broadly mentioned as necessary to design, implement and adapt programmes to achieve best fit for local contexts. Some participants had a research role (i.e. association with a university) which might have explained this thinking, although the importance of embedding research into programme development and its ongoing adaptation to achieve population-level health benefits was prominently discussed by all. Building research into practice was suggested to enable programmes to be continually adapted to meet evolving needs, not just as a token evaluation process. Evaluation and routine data collection were however also discussed by all as necessary to generate tangible evidence of effective PPPs in order to communicate benefits to decision-makers and to sustain funding.

*…The key thing there is you build your health promotion systems, the organizations, the processes, etc., to integrate research into its governance and build it into its recruitment, build it into its training …in this case research is being part of health promotion.* #07 Local health senior manager

*If I have a programme evaluation that is demonstrating a programme to be effective beyond reach …it increases the likelihood for me of either at least protecting it, if not ideally actually increasing or sustaining investment into the long term. That makes a really huge difference compared to a programme that isn’t evaluated.* #11 State NGO area manager

## Relation to existing frameworks

Critical examination of the data against the PSAT framework revealed aspects of PPPs similar to and different from the determinants defined in the existing literature (Additional file 2: Table S1 for qualitative synthesis). As an example, strategic planning was obviously used by participants to “guide programme directions, goals and strategies” as defined [15], although the tension between policies and strategies and agencies across the different levels of the system was also evident—as discussed above. The importance of partnerships, funding stability, leadership, research, support structures, adaptation and communication were all discussed in terms of enabling factors, but diverged from the existing understanding in that participants discussed these factors within the context of interactions between system levels (i.e. between organizations) and in the context of being pathways for interaction (e.g. where priorities, goals or internal processes differed).

## Discussion

Population-delivered programmes play an important role in disease prevention and health promotion [21]. Most of the existing research has focused on how to sustain small-scale interventions in single=organization structures. Fewer studies have identified factors that hinder or facilitate sustainment of PPPs from a complex system level [19, 27], and test the application of existing theories and causal mechanisms across multiple levels [37]. Therefore, we extend the current literature by capturing real-world experiences of policy actors directly involved in delivering PPPs across system levels of influence, and offer important insights for expanding the sustainability literature.

We identified four key factors stakeholders perceived to influence the sustainment of PPPs. These factors represent the challenges in implementing a standardized and rigid “one-size-fits-all” programme across different levels, population settings and structures, and divergent policies and sociopolitical “turbulence” [27]. Two barriers were associated with the changing socioeconomic and political environments—political and funding cycles and competing interests. Changes in government, short budget cycles and internal political pressures are now recognized as “new factors” influencing sustainment [18]. These factors are infrequently mentioned, but population delivery is most susceptible to social, economic and political environmental changes [22]. These findings highlight the gap in current literature and linear understanding of factors influencing programme sustainability. As the literature expands to explore factors relevant to sustaining policies [3, 37–39], these findings are most relevant. Elsewhere, factors of conflicting stakeholder interests and differences across local–regional–state policies and their integration are beginning to be recognized as important for determinants of whole-of-system programmes [10].

A deeper understanding of systems approaches to public health is emerging as the way to address complex health issues that cannot be solved by simple programmes and standardized implementation [40–42]. PPPs implementation and strategies to enable sustainment also need to move towards a whole-of-system approach. Yet the findings described in this study suggests a generally linear downstream approach—from state or national directive to local implementation—in how programmes are designed, implemented and maintained. The COVID-19 pandemic is a current example of systemic interruption of the delivery of many PPPs (e.g. physical activity, healthy eating programmes) and the disruption of the workforce for these programmes by mandated public health guidelines (e.g., physical distancing) and reallocation of health resources [43]. This “perfect storm” has laid bare unintentional consequences of *not* sustaining PPPs when social, political or other economic changes arise. Consequences included diminished mental and physical health across all age groups [44–46]. Therefore, a systems approach to sustain PPPs is needed to buffer against the effects of unanticipated crises such as COVID, which occur during ongoing increasing trends in NCDs. This is the opposite of “silo thinking”, which compartmentalizes the actions of organizations and their short- and long-term contribution to PPP sustainment.

The next step for improving PPP sustainability is to further understand the system. System dynamics modelling is a tool for determining where to intervene to bring about change in the system, such as where and how to intervene to improve sustainability [47]. This approach is used to identify causal chains, such as what is driving the system—for example, what is causing it to produce only short-term programmes, what is causing undue competition for NCD prevention, or contributing to silo thinking or poorly fit programmes. Other systems methods can be applied to better understand the structures which are resulting in certain outcomes or interconnections in the system, including social network analysis and agent-based modelling [48, 49]. Enabling factors for the sustainment of PPPs, including the role of agencies, also need to be better understood from a whole-of-system perspective. Research and evaluation evidence is necessary for understanding and changing the system, and information feedback into programmes can help reveal how a PPP should evolve and adapt in response to changes in actors, environments or contexts [50].

Small solutions were being implemented by participants in this study to support programme sustainment, and there is a need to move beyond simple or easy system modifications to more seismic action and culture change [28]. We identified the need for designing flexible, agile programmes from the start, and employing long-term whole-of-system planning and resourcing, which moves beyond individual programmes to the shifting and emerging population needs, recognizing the evolving nature of programmes and needs over time [37]. Flexible and agile programmes are better able to withstand a changing sociopolitical environment and respond to shifting population needs. But a systems perspective on NCD issues today recognized the complex causality and need for programmes which often address multiple outcomes [31, 48, 49]. This means thinking of individual programmes and their sustainability as part of the combined effort rather than in isolation [48, 49]. When a system is working well, it is showing characteristics of resilience, self-organization or hierarchy [28]. If hierarchy is important for system stability, then it needs to be supported upstream, downstream and cross-stream through a whole-of-system mindset of the various actors and their part in the system to reduce the risk of silo actions and passing of responsibility as the solution to programme sustainment. Self-organization requires freedom to evolve—like the concept of “tight–loose–tight” explained by one participant. Resilience is described as the opposite of rigidity [28]. Adaptation of PPPs is the essence of implementation frameworks [2, 51, 52]. Adaptation to enhance the fit for a population or context is now considered a routine part of programme sustainment [51, 53]. However, the notion of adaptation is contentious, as it gives rise to tension when a PPP deviates from its original [effective] design and implementation approach to meet the needs of a new population or delivery system [37]. Some contend that modifications are necessary [10, 14] and even preferred [53, 54], and while our research concurs, questions remain as to whether PPPs’ “core factors” need to be maintained to retain effectiveness and how to maintain effectiveness in a dynamic, shifting system. The sustainability literature also needs to evolve to encompass systems theories, concepts and lexicon [55] to move beyond the current interest in linear sustainability frameworks and templates.

### Strengths and limitations

A key strength of our study is that it is one of few studies to explore how population health programmes can be sustained at the population level. Participants represented extensive, diverse experiences and real-world insights as to factors that influenced sustaining PPPs. This scope of experience may have contributed to their contemplation of factors at the systems level, whereas most of the current literature addresses how one organization operates or achieves implementation success over shorter periods.

Limitations include our sample size, which was relatively small. It is likely that at the time of data collection, senior public health stakeholders were preoccupied with infectious disease control efforts. Despite this, most ideas were gathered within the first 5–6 interviews, with the next set of interviews providing confirmation and suggesting we had reached a threshold of new information, and the last two interviews supporting this saturation of ideas [54]. Although the sample was stratified by the participants’ roles within government/NGOs, the majority had worked across organizations and provided examples from their various roles and areas of expertise. This may explain the degree to which we found that new data repeated what was expressed in earlier interviews [56]. However, we note that participants were primarily from one state within Australia. Each state has a slightly different governance structure. This limits the capacity to generalize findings to all groups and jurisdictions in Australia and elsewhere. However, themes that arose reflected ideas expressed by participants from other states and confirmed by participant checking of findings.

## Conclusion

The sustainment of population prevention programmes at the population level is fraught with many challenges but is necessary to improve long-term population health outcomes. Factors work independently and in concert within and across numerous levels of a complex, dynamic system. The implications of embedding PPPs into the Australian health system, the need for long-term stable funding in ever-changing sociopolitical environments and funding cycles, and the need to embrace the role of multiple organizations holds relevance for other countries.

## Supplementary Information

**Additional file 1.** Discussion guide.

**Additional file 2: Table S1.** Critical analysis of population prevention interventions in relation to the PSAT domains.

## Data Availability

The datasets generated and/or analysed during the current study are not publicly available due to ethics requirements to ensure privacy of identifying information.

## Abbreviations

CEO: Chief executive officer
PPP: Population prevention programme
NGO: Nongovernmental organization
NSW: New South Wales
NPAPH: National Partnership Agreement on Preventive Health
