# Supplementary material for: Understanding the sustainment of population health programmes from a whole-of-system approach
Source: Health Res Policy Syst. 2022 Apr 7;20:37. doi: 10.1186/s12961-022-00843-0 (PMC8988542; doi:10.1186/s12961-022-00843-0)
Supplement: Supplementary file 1 — Additional file 1. Discussion guide. [file 12961_2022_843_MOESM1_ESM.docx]

**Additional file 1 – Discussion guide**

| Area of interest | | | | Potential questions |
| --- | --- | --- | --- | --- |
| Topic: Background and involvement in Intervention | | | | |
| Current role & previous experience in sustaining interventions | | | | Can you tell me a bit about your experience in respect to large scale population interventions that have been sustained? In what capacity were you involved? What were the type of responsibilities you had? By large scale – I mean population wide interventions. By sustained I mean continues to be delivered for at least 12 months beyond cessation of active implementation support.  What interventions come to mind? How long have they been sustained?  How long would you consider an intervention needs to be in operation before you can call it ‘sustained’?  What needs to be put in place before you think an intervention is likely to be sustained? |
| Topic: overview | | | | |
| General overview:  How do you sustain a population intervention? | | | Thinking about any public health initiatives that you were responsible for that was sustained.   1. Briefly, what are some of the challenges that you have experienced to sustain the ongoing delivery of an intervention? <prompt examples> 2. Have you managed the delivery/implementation of interventions through a change in government or change in government priorities? How did you do that?   What was the situation? What was the initiative and how did you overcome any barriers? Did it lead to new opportunities?   1. Have there been other social or political changes you have gone through to sustain the program? [prompt *i.e. change in social or economic environment (e.g. COVID) change in technology? (i.e. digitalisation)].* How did that either enable or inhibit the sustainment of the program? 2. What other types of situations or challenges are you aware of that have affected the sustainability of interventions you have been responsible for? (i.e. voltage drop – where the benefits drop, program shift) How have you overcome these challenges? 3. What do you think has been the key thing that enables program(s) to be sustained? If multiple initiatives then has this been true for other programs you were responsible for? | |
| Topic: Partnerships | | | | |
|  | I’m now going to ask you about the role partnerships in sustaining any of these programs. These partnerships could be internal or external to your organisation (can include gov or NGO, industry partners).   1. How important are partnerships for sustainment of your organisation’s population interventions [prompt describe any partnerships] 2. What sort of partnerships do you look for? Do you have sustainability in mind when looking for partners? [with similar organisations, with industry/private-public partners, government or NGO?] 3. What was the role of partners in sustaining the intervention (i.e. services delivery, design, shared/manage, co-fund, funding capacity, resources or training?) 4. Have partnerships been affected by the change in government, gov priorities or socio/political situation you mentioned? How were partnerships maintained (if maintained)? 5. Is there anything else you would like to add about partnerships and their role in enabling, inhibiting program sustainment | | | |
| Topic: Socio-political support | | | | |
|  | Thinking about political or social support of the intervention(s)   1. We discussed some of the challenges of social-political changes. Can you think of any social or political support you have had (or didn’t have) that has influenced the sustainability of a program or initiative? Explain. 2. Were there potential people / decision makers that you needed to win over (policymakers, state legislators, elected officials) to ensure the program’s sustainability? Or other stakeholder groups that you have had to partner with in order to reach different decision makers? 3. Were there any external champions that you use(d) to promote the values of the intervention? If so how do you ensure their ongoing interest? | | | |
| Topic: Funding stability | | | | |
|  | | Now thinking in terms of funding support of the program   1. Is ongoing funding necessary for sustaining the program? How so? <ref to institutionalisation where funding becomes routine> 2. What types of funding support have you relied on and what impact has that had on the capacity to sustain the intervention(s) (line-item budgets, individual/business donors, national/state grants, fundraising, fee-for-service)? 3. Can you describe a situation where funding stability for a successful intervention was at risk? What did you do to maintain funding? Did the funding structure change? What implications did this have on the program/how was it affected? 4. What would you say is necessary to do in order to create/ maintain funding stability? 5. Is there anything else you would like to add about the role of funding? | | |
| Topic: Strategic planning & organisational capacity | | | | |
|  | | Now thinking in terms of strategic planning and your organisation’s capacity   1. Was/is program sustainability planned from the outset? How? <is it included in a scalability plan or just evolves> 2. Who makes decisions on whether it is sustained or not - program manager alone/ senior leadership etc?   [if managed by 3^rd^ party] How do you plan and manage the sustainability of an intervention? What is your organisation’s capacity for sustainability in terms of organisational structures and processes, internal policies? (e.g. to ensure sufficient core staff expertise & skills?)   1. What organisational learning (problem solving capacity) is used to help the interventions longevity (ongoing assessment, feedback learning)? | | |
| Topic: Program adaptation | | | | |
|  | | Thinking about interventions that have been running for a few years,   1. What sort of adaptations do you make to the intervention at this stage? Do these adaptations tend to be planned or unplanned? 2. Who is it who decides to modify the intervention (program manager, funder, stakeholders, CEO)? 3. Thinking about specific intervention, what sort of changes are made to interventions to ensure their longevity and why (content, context, delivery system, training)? | | |
| Topic: Program evaluation | | | | |
|  | 1. What research and evaluation methods do you use to inform the sustainability of interventions? 2. How has research and evaluation helped to either support change or initiate change in the intervention or its delivery?’ 3. Are researchers or university organisations ever involved in investigating the outcomes or impacts of your interventions? In what circumstances? 4. What sort of information and how frequently is it collected to improve interventions after they have been in operation a few years? (e.g. info on the target pop, awareness, satisfaction, env climate) | | | |
| Topic: Communication | | | | |
|  | | | 1. Do you have a formal communication plan to assist in the program’s sustainability? 2. What communication strategies do you use to improve the program’s longevity? i.e. ensure organisational leadership, stakeholders, funders or public are aware of the program’s outcome and impacts. | |
| Close | | | | What are some of the key learnings that could be drawn from this experience to inform future sustainment of population health initiatives?  Do you have any final thoughts on anything we have not discussed? |

*the sub-topics were based upon the Program Sustainability Assessment Tool (PSAT). (Schell, Luke et al. 2013)
