# Supplementary material for: Understanding the sustainment of population health programmes from a whole-of-system approach
Source: Health Res Policy Syst. 2022 Apr 7;20:37. doi: 10.1186/s12961-022-00843-0 (PMC8988542; doi:10.1186/s12961-022-00843-0)
Supplement: Supplementary file 2 — Additional file 2: Table S1. Critical analysis of population prevention interventions in relation to the PSAT domains. [file 12961_2022_843_MOESM2_ESM.docx]

**Additional file 2.**

**Table S1 critical analysis of population prevention interventions in relation to the PSAT domains^.**

| **PSAT item** | **PSAT definition** | **What agreement the research makes with this factor?** | **What was its relevance for sustaining interventions at the population scale?** | **Example quotes** |
| --- | --- | --- | --- | --- |
| **Strategic planning** | *Using processes that guide your program’s directions, goals, and strategies* | Key performance indicators & monitoring management systems other data, & service agreements help support planning | Population scale requires longer-term planning than current practice, & not just in internal planning but requires system-wide strategic planning to consider where priorities with other agencies coalesce & conflict. | *some of the policies are working together.* [where it doesn’t] …*those cross-agency partners I think are going to be difficult. Because if you've got policy directives that butt up against each other, how do you resolve that?* |
| **Environmental support** | *Having a supportive internal and external climate for your program.* | Internal support was mentioned in terms of leadership & champions. External support included public profile or public reputation | Both internal support & external public support have relevance, and should both be utilised to withstand socio-political changes | *keeping an eye on what’s in the public discourse and sentiment or the support … therefore you have permission to act* |
| **Funding stability** | *Establishing a consistent financial base for your program.* | Funding stability helps but cannot be relied upon. Funding was generally discussed in connection with other resourcing options (i.e. if no funding then look to resource differently or creatively to make it happen). | While funding is necessary, it’s finite so need to be used strategically (creative resourcing, embedding policies partnerships agreements & organisational support). | *the one thing that really has sustained them all is the funding agreements that we have for them*. |
| **Partnerships** | *Cultivating connections between your program and its stakeholders.* | Fostering partnerships was generally considered an essential mechanism for sustaining PPPs or keeping them afloat. | Partnerships are part of the framework supporting PPPs. At all levels, partnerships are utilised to enable program delivery, reduce resourcing & other costs, or distribute environmental risks. | *what they do is they provide political imprimatur; they provide a framework into which your specific initiative or program might comfortably sit.* |
| **Organisational capacity** | *Having the internal support and resources needed to effectively manage your program.* | Internal capacities required were generally stretched, knowledge loss a reality & required novel flexible approaches to harness capabilities & resources | Requires a system of organisations as single organisation capabilities, knowledge or resources are unable to achieve population reach & impact | *we can't do it on our own - …so we actually tend to capacity build others to then make things happen.* |
| **Program evaluation** | *Assessing your program to inform planning and document results* | Evaluation & monitoring were central to inform planning & defend programs against funding cuts | Evidence of intervention impact & reach across the population requires implementing systems measures to capture data and investigate differences across localities | *that monitoring data helped us to be able to talk about the impacts in a way that was very tangible… to show reach and impact in a way that we hadn’t been able to do before* |
| **Program adaptation** | *Taking actions that adapt your program to ensure its ongoing effectiveness.* | The need for ongoing adaptation was considered essential as part of the lifecycle of programs but is generally reactive | Adaptation of programs to the needs of the population means having both the flexibility & systems in place to enable adaptations to fit with needs | *There’s kind of like a shelf life of programs, and it could be anywhere between five and 10 years. But at some point, it gets a bit stale, tired, people are familiar with it, think they’ve done it, and need something new.* |
| **Communications** | *Strategic communication with stakeholders and the public about your program* | Communication with stakeholders about the program’s impacts was necessary to promote the program; a few also commented how a program’s public reputation could assist | Communication to stakeholders & public were essential. Good upstream communication was perceived as hard work, but also as a necessary set of skills. | *if you don’t have the right communication skills and the right influencing skills it makes those relationships that you have with senior executive, or with the political powers that be, so much harder*. |

^ Program Sustainability Assessment Tool (PSAT). (Schell, Luke et al. 2013)

Schell, S. F., D. A. Luke, M. W. Schooley, M. B. Elliott, S. H. Herbers, N. B. Mueller and A. C. Bunger (2013). "Public health program capacity for sustainability: a new framework." Implementation Science **8**(1): 15.
